# Tryptophan metabolism in tumor immune escape: mechanisms, cellular crosstalk, and therapeutic opportunities

**DOI:** 10.3389/fimmu.2026.1878140

**Published:** 2026-07-08

**Authors:** Jingyun Chen, Xin Wang, Meiheng Gong, Yufeng Wang, Tingting Yu

**Affiliations:** 1 Cancer Institute, The First Hospital of Jilin University, Changchun, China; 2 Department of Otolaryngology Head & Neck, The First Hospital of Jilin University, Changchun, China

**Keywords:** immunometabolism, kynurenine, tryptophan metabolism, tumor immune escape, tumor microenvironment

## Abstract

Tumor immune escape is increasingly recognized as an immunometabolic process shaped not only by immune checkpoints and suppressive cell populations, but also by nutrient competition and metabolic signaling within the tumor microenvironment. This nutrient-competitive environment is not limited to tryptophan depletion, but also involves glucose restriction, glutamine dependence, arginine metabolism, amino acid transporter competition, and impaired mitochondrial fitness of effector T cells. Among amino acid pathways, tryptophan metabolism has emerged as a central regulator of tumor–immune interactions. Through the activity of indoleamine 2, 3-dioxygenase 1 (IDO1), tryptophan 2, 3-dioxygenase (TDO2), kynurenine-producing branches, and both AHR-dependent and AHR-independent downstream programs, tumors establish a metabolic state that couples tryptophan depletion, metabolite signaling, redox adaptation, and immune suppression. Recent evidence further shows that tryptophan metabolism is not restricted to tumor cells, but also involves cancer-associated fibroblasts, macrophages, and T cells, thereby shaping multicellular crosstalk within immunosuppressive niches. Beyond immune suppression, this pathway contributes to ferroptosis resistance, stemness, metastatic adaptation, and resistance to chemotherapy, targeted therapy, and immune checkpoint blockade. In parallel, circulating metabolites and tissue-level metabolic profiling are being explored as potential biomarkers for patient stratification and treatment response prediction. In this review, we summarize the molecular basis of tryptophan catabolism in cancer, discuss its role in tumor–immune–stromal communication, and highlight emerging translational and therapeutic opportunities. Rather than reviewing tryptophan metabolism as a linear IDO1/TDO2-centered pathway, we define it as a multicellular immunometabolic communication network in which tumor cells, stromal fibroblasts, myeloid cells, and lymphocytes exchange metabolic and signaling cues to create spatially organized immunosuppressive niches. This network-based view helps explain why single-enzyme inhibition is often insufficient and supports the development of biomarker-guided, multi-branch, and cell-context-specific therapeutic strategies.

## Highlights

- Tryptophan metabolism drives tumor immune escape through coordinated effects on tryptophan depletion, kynurenine signaling, and AHR activation.
- This pathway reshapes tumor–immune–stromal crosstalk by regulating macrophage polarization, CAF-mediated immune exclusion, and CD8+ T-cell dysfunction.
- Beyond immune suppression, tryptophan metabolism promotes ferroptosis resistance, metastasis, therapy resistance, and offers opportunities for biomarker-guided combination treatment.

## 1 Introduction

Tumor immune escape has traditionally been interpreted through the lens of immune checkpoints, suppressive cytokines, regulatory T cells, and myeloid-derived inhibitory populations (1–5). These mechanisms remain central to our understanding of how malignant cells evade immune surveillance, yet they do not fully explain the persistent failure of antitumor immunity across diverse cancer types. Increasing evidence indicates that immune escape is not solely an immunological phenomenon, but also a metabolic one. Tumors evolve within nutrient-restricted microenvironments in which malignant cells, stromal cells, and immune cells compete for essential metabolites while simultaneously reshaping the biochemical landscape through altered metabolic activity (6–9). In this context, metabolic reprogramming is not merely a consequence of tumor progression; it is an active determinant of immune dysfunction.

Among the different metabolic programs implicated in cancer, amino acid metabolism has emerged as a particularly important regulator of immune behavior. Amino acids are not only substrates for protein synthesis and bioenergetic adaptation, but also signaling molecules that influence cell fate, immune activation, differentiation, and stress responses (10–13). Tumor-driven disturbances in amino acid availability can therefore exert profound effects on the quality and durability of antitumor immunity (14–16). Rather than acting as passive bystanders, metabolic pathways shape whether immune cells retain cytotoxic competence, adopt exhausted phenotypes, or are diverted toward immunoregulatory states. Within this broader immunometabolic framework, tryptophan metabolism has attracted special attention because it operates at the interface of nutrient deprivation, metabolite signaling, and transcriptional reprogramming (17–19). Accelerated tryptophan catabolism in the tumor microenvironment can deplete local tryptophan pools, thereby limiting T-cell proliferation and effector function, while simultaneously generating bioactive metabolites that actively suppress antitumor immunity (20, 21). These metabolites, especially kynurenine, engage signaling pathways such as the aryl hydrocarbon receptor (AHR), which further promote immune tolerance, tumor adaptation, and therapeutic resistance (22–24). Thus, tryptophan metabolism should not be viewed as a simple metabolic abnormality, but rather as a coordinated immunometabolic axis that links tumor cell survival to immune evasion. Importantly, the relevance of tryptophan metabolism extends beyond mechanistic biology. Growing evidence suggests that this pathway contributes to immune-cell dysfunction, stromal remodeling, metastatic progression, and resistance to systemic therapy, including immune checkpoint blockade (25–28). Accordingly, understanding tumor immune escape now requires a broader conceptual shift: from viewing metabolism as a background feature of cancer to recognizing it as an organizing force that governs tumor–immune interactions.

Although multiple amino acid pathways are involved in tumor progression and immune regulation, tryptophan metabolism deserves particular emphasis because of its unusually well-defined mechanistic framework and broad biological reach. A major reason is the existence of a canonical and highly tractable signaling axis centered on indoleamine 2, 3-dioxygenase 1 (IDO1), tryptophan 2, 3-dioxygenase (TDO2), kynurenine, and AHR (29–32). This axis provides a coherent molecular model through which changes in nutrient metabolism are translated into immune suppression and malignant adaptation. In contrast to pathways that are metabolically important but mechanistically diffuse, tryptophan catabolism offers a clear chain of events linking enzyme induction to metabolite accumulation, receptor activation, and downstream transcriptional programs.

A second reason is that the biological influence of tryptophan metabolism is not confined to tumor cells. Rather, it acts across multiple cellular compartments within the tumor microenvironment. Tumor cells can exploit tryptophan catabolism to support their own survival and immune resistance, but the same pathway also reshapes the behavior of nonmalignant stromal and immune populations. Cancer-associated fibroblasts may amplify immunosuppressive signaling and restrict cytotoxic lymphocyte infiltration. Macrophages may be reprogrammed toward tumor-promoting and immunoregulatory phenotypes (33, 34). T cells may undergo proliferative arrest, functional exhaustion, or exclusion from the tumor bed. This multicellular scope makes tryptophan metabolism particularly suitable for understanding immune escape as a systems-level process rather than a tumor cell-intrinsic event. Third, tryptophan metabolism has unusually strong translational relevance. Recent studies connect this pathway not only to immune suppression, but also to metastasis, stemness, therapeutic resistance, and response heterogeneity in patients receiving immunotherapy. In addition, circulating metabolites such as the kynurenine/tryptophan ratio have been explored as potential blood-based biomarkers, while tissue-level expression of IDO1, TDO2, and related metabolic programs may help define immunosuppressive niches within tumors (35, 36). These clinical associations make tryptophan metabolism more than a mechanistic curiosity; they position it as a candidate axis for patient stratification, biomarker development, and therapeutic intervention. Taken together, tryptophan metabolism deserves special attention because it combines conceptual clarity, multicellular relevance, and translational value. It provides a framework through which the metabolic ecology of the tumor microenvironment can be connected to immune dysfunction, disease progression, and treatment failure.

The conceptual advance of this review is to move beyond the conventional enzyme-centered model of tryptophan metabolism. Previous reviews have largely focused on IDO1 or TDO2 induction, tryptophan depletion, kynurenine accumulation, AHR activation, and subsequent T-cell suppression (18, 22). Although this linear model remains fundamental, it does not fully explain several clinically relevant observations, including spatial immune exclusion, stromal amplification of immune suppression, myeloid reprogramming, metastatic niche formation, ferroptosis resistance, and the limited efficacy of single-node IDO1 inhibition (37–40). We therefore define tryptophan metabolism as a multicellular immunometabolic communication network with three key features. First, pathway activity is distributed across tumor cells, CAFs, macrophages, dendritic cells, MDSCs, Tregs, NK cells, and CD8+ T cells rather than being restricted to malignant cells (41–43). Second, its biological output is spatially organized into suppressive niches characterized by kynurenine-rich signaling, myeloid enrichment, stromal remodeling, checkpoint activation, and impaired T-cell infiltration. Third, its therapeutic relevance is shaped by pathway redundancy, metabolic branching, and non-enzymatic IDO1 functions, which may preserve tumor-promoting outputs even when a single enzymatic node is blocked (37, 38, 44, 45). This framework is mechanistically important because it links nutrient deprivation, metabolite signaling, cellular crosstalk, tissue spatial organization, and treatment failure into one integrated model. It also changes therapeutic logic from simple IDO1/TDO2 inhibition toward biomarker-guided, multi-branch, and cell-context-specific intervention.

In this review, we use this network-based framework to examine how tryptophan metabolism coordinates tumor immune escape, stromal remodeling, myeloid reprogramming, metastatic adaptation, and therapeutic resistance. We first summarize the core molecular architecture of tryptophan catabolism, with particular emphasis on the IDO1/TDO2–kynurenine–AHR axis and its role in coupling metabolic reprogramming to immunosuppressive signaling. We then discuss how this pathway operates across different cellular compartments of the tumor microenvironment, focusing on the crosstalk among tumor cells, cancer-associated fibroblasts, macrophages, and T cells. Particular attention is given to the immunological consequences of this metabolic rewiring, including suppression of CD8+ T-cell activity, macrophage polarization toward tumor-supportive states, and CAF-mediated immune exclusion. We further extend the discussion beyond classical immune suppression to highlight emerging evidence linking tryptophan metabolism to ferroptosis resistance, metastatic adaptation, stemness, and broader forms of therapeutic resistance. In addition, we review the growing translational literature on circulating and tissue-based biomarkers related to tryptophan metabolism, as well as their potential utility in predicting immunotherapy response. Finally, we discuss current and emerging therapeutic strategies aimed at targeting tryptophan metabolism in cancer. Rather than focusing exclusively on single-enzyme inhibition, we consider the broader challenge of rewiring an immunometabolic network that spans tumor, immune, and stromal compartments. By integrating molecular mechanisms, cellular crosstalk, and translational opportunities, this review aims to provide a comprehensive framework for understanding how tryptophan metabolism shapes tumor immune escape and how this knowledge may inform future therapeutic development.

## 2 Core molecular basis of tryptophan metabolism in cancer immune escape

**Table 1** summarizes the core molecular components of tryptophan metabolism and highlights how this pathway couples immunosuppressive signaling to tumor-promoting adaptation.

**Table 1:** Core molecular components and biological consequences of tryptophan metabolism in tumor immune escape.

Component/axis	Main cellular source	Major downstream effect	Immunological consequence	Tumor-promoting consequence	Representative studies
IDO1	Tumor cells, immune cells	Accelerated tryptophan degradation	T-cell suppression, immune tolerance	Supports immunosuppressive TME	Shu 2024 (83); Liang 2022 (65)
TDO2	Tumor cells, CAFs, stromal fibroblasts	Kynurenine production	CD8+ T-cell dysfunction, immune escape	Promotes stemness, metastasis, resistance	Miyazaki 2022 (57); Li F 2021 (58); Liu Y 2024 (39); Lu 2025 (40)
Kynurenine	Tumor and stromal metabolic product	Bioactive metabolite signaling	T-cell dysfunction, macrophage reprogramming	Enhances tumor adaptation and survival	Miyazaki 2022 (57); Liu Y 2024 (39)
AHR	Tumor cells, immune cells, stromal cells	Transcriptional reprogramming	Immunoregulatory signaling	Stemness, therapy resistance, malignant plasticity	Li F 2021 (58); Feng 2022 (78); Miyazaki 2022 (57)
Branched Trp metabolism (Kyn/indole/5-HT pathways)	Tumor cells and TME	Multiple metabolic outputs	Sustains immune suppression through parallel routes	Favors therapy resistance and metabolic plasticity	Li J 2024 (38)
Trp metabolism–ferroptosis axis	Tumor cells, metastatic niche	Anti-ferroptotic adaptation	Indirectly sustains immune escape	Enhances survival and metastatic persistence	Liu Y 2024 (39)

### 2.1 Canonical tryptophan catabolism: IDO1, TDO2, kynurenine, and AHR

At the core of tryptophan metabolism in cancer lies a canonical catabolic axis centered on indoleamine 2, 3-dioxygenase 1 (IDO1), tryptophan 2, 3-dioxygenase (TDO2), kynurenine (Kyn), and the aryl hydrocarbon receptor (AHR) (46–49). This pathway has become one of the best-characterized examples of how metabolic reprogramming can be translated into immune suppression and tumor adaptation. In physiological contexts, tryptophan catabolism contributes to immune homeostasis and tolerance. In cancer, however, this machinery is frequently co-opted by malignant cells and the surrounding microenvironment to create conditions that are unfavorable for effective antitumor immunity. IDO1 and TDO2 catalyze the initial and rate-limiting steps of tryptophan degradation along the kynurenine pathway (50–52). Their upregulation in tumors leads to accelerated local consumption of tryptophan and accumulation of kynurenine and related metabolites. This metabolic shift has two major consequences. First, depletion of extracellular tryptophan creates a nutrient-restricted environment that compromises lymphocyte proliferation, activation, and effector function. T cells are particularly sensitive to amino acid insufficiency, and chronic tryptophan deprivation can contribute to impaired cytotoxicity, proliferative arrest, and defective immune surveillance. Second, kynurenine is not a passive byproduct of catabolism but an active signaling metabolite that exerts broad immunoregulatory and tumor-promoting effects.

A central downstream mediator of kynurenine signaling is AHR, a ligand-activated transcription factor increasingly recognized as a metabolic sensor in cancer. Once activated by kynurenine, AHR drives transcriptional programs that extend far beyond detoxification responses (53–56). In tumor settings, AHR activation has been linked to immune tolerance, altered cytokine programs, increased tumor cell plasticity, and reinforcement of malignant phenotypes. Thus, in cancer, tryptophan catabolism is not merely a metabolic sink but a signaling axis that couples nutrient depletion to kynurenine–AHR-dependent immunosuppressive and tumor-promoting programs.

Several recent studies illustrate how this canonical pathway functions in specific tumor contexts. Miyazaki et al. showed that the TDO2–Kyn–AHR axis contributes to immune evasion and metastatic behavior in colon cancer, linking kynurenine signaling to PD-L1 expression, stemness-related traits, and liver metastasis (57). Mechanistically, this study links TDO2–AHR activation to both checkpoint-associated immune escape and metastatic competence, supporting the idea that immune suppression and tumor adaptation can be co-organized by the same metabolic axis. Likewise, Li et al. reported in prostate cancer that TDO2-driven kynurenine production promotes chemotherapy resistance through an AHR/c-Myc-dependent mechanism, providing a clear example of how tryptophan metabolism supports tumor-intrinsic malignant adaptation in addition to immune suppression (58).

The pathway is also subject to upstream regulation by tumor-specific drivers. Shu et al. identified PABPC1L as an upstream factor that induces IDO1 in renal cell carcinoma and thereby promotes tryptophan metabolism and immune suppression. This finding is mechanistically important because it shifts the discussion from enzyme overexpression alone to the broader regulatory architecture that sustains metabolic immune escape. It suggests that IDO1 activation may be embedded within oncogenic or post-transcriptional networks that enable tumors to stabilize an immunosuppressive metabolic phenotype. Another important concept emerging from this literature is functional redundancy within the tryptophan-catabolic system. Wu et al. demonstrated in platinum-resistant non-small cell lung cancer that dual inhibition of IDO1 and TDO2 more effectively enhances antitumor immunity than targeting either enzyme alone (37). This work suggests that tumors may preserve kynurenine pathway activity through compensatory use of parallel enzymes, thereby limiting the efficacy of single-node interventions. Accordingly, the canonical model of tryptophan catabolism should not be interpreted as a simple one-enzyme, one-product cascade, but rather as a flexible metabolic signaling module with built-in adaptive capacity.

Taken together, the IDO1/TDO2–kynurenine–AHR axis remains a central and well-supported model for understanding how tryptophan metabolism contributes to tumor immune escape. However, this canonical axis should be interpreted as an entry point rather than a complete explanation of tryptophan-driven tumor biology. Different tumor types may rely on distinct combinations of AHR activation, amino acid stress responses, redox adaptation, NAD^+^-related metabolism, cytokine remodeling, and stromal or myeloid signaling. Therefore, subsequent sections discuss both AHR-dependent and AHR-independent mechanisms to avoid reducing a heterogeneous metabolic pathway to a single downstream receptor.

### 2.2 Beyond tryptophan depletion: metabolite signaling and pathway branching

Although tryptophan depletion has long been considered a central mechanism of tumor-associated immune suppression, this view alone is no longer sufficient to explain the biological reach of tryptophan metabolism in cancer. A more contemporary understanding recognizes that the functional consequences of this pathway arise not only from substrate loss, but also from the generation of bioactive metabolites and the existence of multiple downstream branches with distinct cellular effects. In other words, tryptophan metabolism should be regarded not simply as a process of amino acid exhaustion, but as a dynamic signaling network.

Among the metabolites generated through tryptophan catabolism, kynurenine is the most intensively studied and remains the dominant immunoregulatory mediator in current cancer literature (59–63). Rather than acting as a passive waste product, kynurenine serves as a signaling molecule capable of reshaping cellular behavior in both malignant and nonmalignant compartments. Through AHR activation, kynurenine can promote transcriptional programs that weaken antitumor immune responses while also enhancing tumor cell survival, plasticity, and adaptation to stress. This dual role is important because it shows that tryptophan metabolism is capable of coordinating tumor-extrinsic immune suppression with tumor-intrinsic malignant fitness. The study by Li et al. in prostate cancer provides a strong example of this principle. Their work demonstrated that TDO2-induced kynurenine activates AHR and downstream c-Myc-associated programs, thereby promoting chemotherapy resistance. This finding underscores that kynurenine signaling is not restricted to suppressing immune cells; it can also directly support oncogenic reprogramming within cancer cells themselves. Such evidence argues against a narrow interpretation of tryptophan catabolism as merely a starvation signal for T cells.

Additional studies further broaden this view by linking tryptophan metabolites to stress resistance and survival pathways. Liu et al. reported that tryptophan metabolism can function as a previously unrecognized anti-ferroptotic pathway that supports tumor growth. This observation is conceptually important because it expands the relevance of tryptophan metabolism beyond immune escape into the realm of cell death control and metabolic resilience. It suggests that metabolites derived from tryptophan catabolism may help tumors withstand oxidative and lipid peroxidation-associated stress, thereby contributing to disease progression even in contexts where immune suppression is not the only selective pressure.

Recent work also indicates that tryptophan metabolism is more branched and interconnected than the classical kynurenine-centered model implies. Li et al. showed in ovarian cancer that amitriptyline can restore immune checkpoint blockade responsiveness by dually inhibiting the Kyn/indole branch and the 5-hydroxytryptamine (5-HT) branch of tryptophan metabolism (38). This study is especially valuable for review writing because it highlights a shift in the field: tryptophan metabolism should no longer be viewed as a linear pathway leading only to kynurenine accumulation, but rather as a network of interacting metabolic routes that together shape tumor behavior and therapeutic response. This broader perspective has two major implications. First, AHR remains a central downstream node, but it is unlikely to account for all the biological consequences of altered tryptophan metabolism. AHR-independent mechanisms are particularly important for a more balanced interpretation of tryptophan metabolism. Downstream kynurenine-pathway metabolites such as 3-hydroxykynurenine, kynurenic acid, xanthurenic acid, and quinolinic acid may influence tumor biology through redox regulation, mitochondrial metabolism, inflammatory signaling, and cellular stress responses. In this context, quinolinic acid is especially relevant because it links tryptophan catabolism to *de novo* NAD^+^ biosynthesis, thereby connecting the kynurenine pathway to bioenergetic adaptation, oxidative stress resistance, and DNA repair capacity. These effects cannot be fully explained by AHR activation alone. Moreover, some biological consequences attributed to “kynurenine signaling” may actually reflect broader metabolic rewiring, including altered NAD^+^ availability, changes in reactive oxygen species handling, ferroptosis sensitivity, and mitochondrial fitness. Therefore, AHR should be regarded as a major but context-dependent node within a wider metabolic network rather than as a universal executor of tryptophan metabolism.

**Figure 1:**
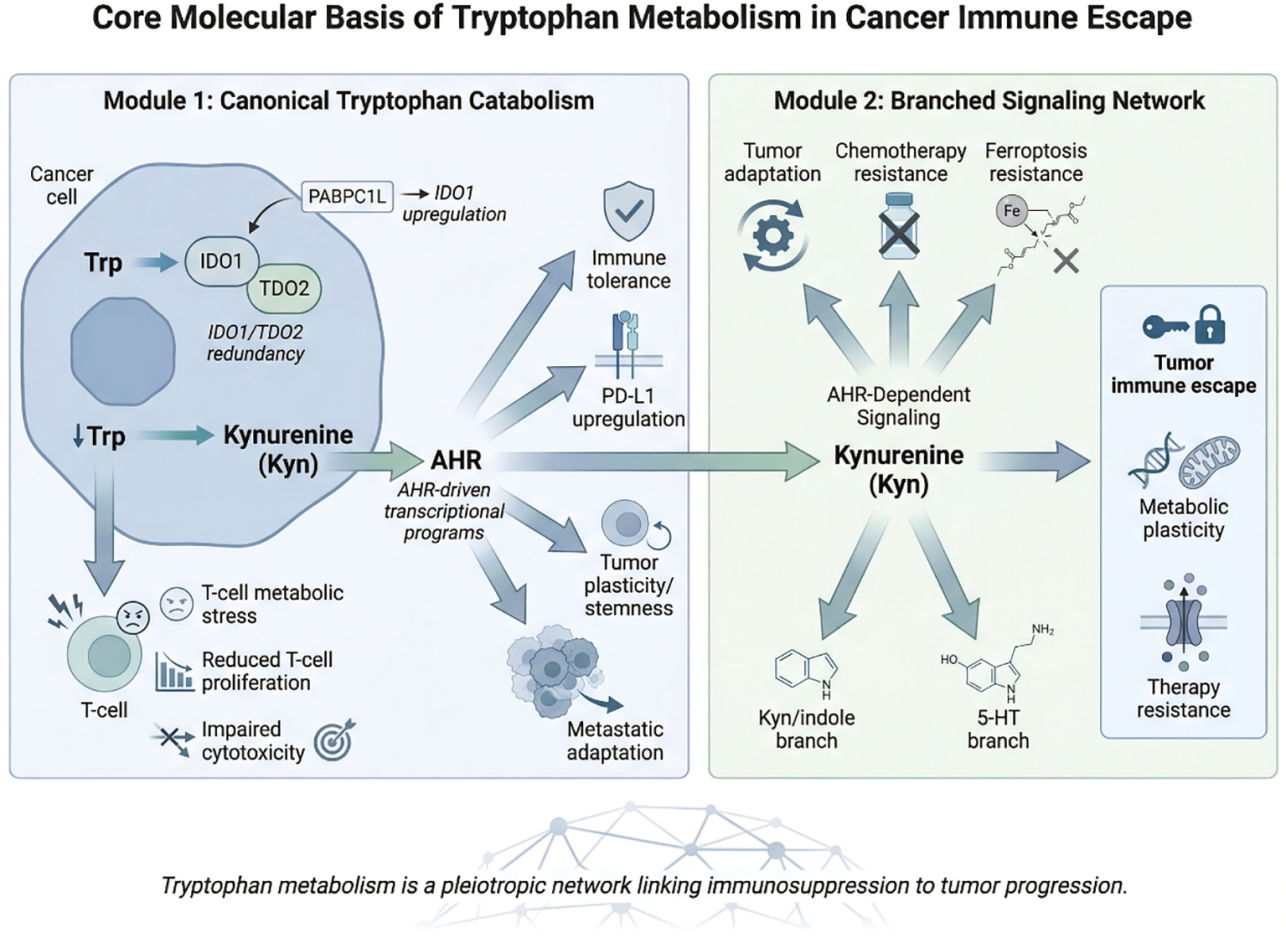
Core molecular basis of tryptophan metabolism in cancer immune escape. Cancer-associated tryptophan metabolism is centered on the IDO1/TDO2–kynurenine–AHR axis, which couples tryptophan depletion to metabolite-driven signaling. Beyond suppressing T-cell function, this pathway promotes immune tolerance, PD-L1 upregulation, stemness, ferroptosis resistance, metastatic adaptation, and therapy resistance through a branched immunometabolic network. This image was created using Adobe Illustrator.

These findings support a branched view of tryptophan metabolism, in which kynurenine-centered signaling represents one important route among several metabolically and therapeutically relevant branches. **Figure 1** illustrates the core molecular architecture of tryptophan metabolism in cancer and highlights how the IDO1/TDO2–kynurenine–AHR axis links nutrient depletion to immunosuppressive and tumor-promoting programs. It also emphasizes that tryptophan metabolism functions as a branched adaptive network rather than a simple linear catabolic pathway.

### 2.3 Mechanistic hierarchy and evidence strength of tryptophan-driven immune escape

A network-based mechanistic hierarchy is useful for separating established core mechanisms from emerging, context-dependent extensions of tryptophan metabolism (18, 64). At the first level are upstream inducers, including inflammatory cytokines, oncogenic signaling, hypoxia, microbial products, stromal cues, carcinogen exposure, and therapy-induced stress, which induce IDO1, TDO2, or related metabolic programs in tumor and non-tumor compartments. At the second level are metabolic mediators, including tryptophan depletion, kynurenine accumulation, indole-related metabolites, 5-HT-associated branches, and other downstream products (65, 66). At the third level are signaling and metabolic nodes, among which AHR is a major but context-dependent transcriptional mediator rather than a universal executor. In parallel, amino acid stress responses, GCN2 activation, mTOR suppression, redox regulation, NAD^+^ biosynthesis, mitochondrial adaptation, cytokine circuits, and non-enzymatic IDO1 signaling may also contribute to tryptophan-driven immune escape and tumor adaptation (67, 68). At the fourth level are cellular targets, including CD8+ T cells, Tregs, NK cells, dendritic cells, macrophages, MDSCs, CAFs, and tumor cells (41, 43, 69). At the fifth level are tissue-level outputs, including immune exclusion, myeloid-enriched suppressive niches, stromal remodeling, ferroptosis resistance, stemness, metastatic adaptation, and therapy resistance (37, 40, 70). This hierarchy clarifies that the strongest evidence currently supports the IDO1/TDO2–kynurenine–AHR axis and its suppressive effects on T-cell function, whereas CAF-mediated immune exclusion, macrophage reprogramming, branched tryptophan metabolism, ferroptosis resistance, metastatic niche adaptation, and non-enzymatic IDO1 signaling represent emerging or tumor-type-dependent mechanisms. Therefore, the proposed network model is not intended to replace the canonical pathway, but to organize it within a broader multicellular and spatial framework that better explains therapeutic failure and guides rational combination strategies.

### 2.4 Tryptophan metabolism within the broader nutrient ecology of the tumor microenvironment

Tryptophan metabolism should be interpreted within the broader nutrient ecology of the tumor microenvironment rather than as an isolated amino acid-depletion pathway (12, 71). Tumor cells, stromal cells, and immune cells compete for multiple shared nutrients, and the functional state of antitumor lymphocytes is shaped by the combined availability of glucose, glutamine, arginine, tryptophan, oxygen, and amino acid transport capacity (8, 12, 72). In this context, tryptophan depletion represents one layer of metabolic pressure that cooperates with other nutrient limitations to impair T-cell activation, proliferation, cytokine production, and cytotoxicity (12, 18). Glucose competition provides a useful parallel. Highly glycolytic tumor cells can restrict glucose availability for infiltrating T cells, thereby reducing mTOR activity, glycolytic capacity, and IFN-γ production (72). This mechanism shows that nutrient competition is not merely a passive consequence of tumor growth, but an active determinant of immune escape. Glutamine metabolism adds another layer of complexity. Tumor cells often depend on glutamine for biosynthesis, redox balance, and mitochondrial metabolism, whereas glutamine availability also influences immune-cell activation and function. Therapeutic glutamine blockade can suppress tumor metabolism while allowing immune cells to adopt compensatory metabolic programs, suggesting that metabolic competition is not always uniformly detrimental to antitumor immunity.

Arginine metabolism further illustrates how amino acid depletion intersects with myeloid immunosuppression (73). ARG1-expressing myeloid cells and MDSCs can reduce local arginine availability, impairing T-cell receptor signaling and effector function (74, 75). This mechanism parallels tryptophan depletion but is often driven by distinct myeloid populations and inflammatory cues. Therefore, tryptophan metabolism should be considered alongside arginine catabolism as part of a broader amino acid-restrictive suppressive niche. Amino acid transporter competition is another important but underappreciated dimension. T cells require coordinated uptake of amino acids through transporters such as SLC7A5/LAT1 and related systems to sustain mTORC1 activation, biosynthesis, and effector differentiation. Tumor cells can exploit the same transport systems or increase amino acid demand, thereby reshaping local nutrient availability (12, 76). In this setting, the effect of tryptophan metabolism may depend not only on enzymatic degradation by IDO1 or TDO2, but also on transporter expression, substrate competition, and the ability of T cells to maintain amino acid uptake under nutrient stress (77).

Finally, nutrient competition converges on mitochondrial fitness. Effective CD8+ T cells require adequate mitochondrial function, redox balance, and metabolic flexibility to sustain cytotoxic activity in nutrient-poor and hypoxic tumor regions. Tryptophan depletion, glucose restriction, glutamine limitation, arginine catabolism, and suppressive metabolites may collectively impair mitochondrial adaptation and promote T-cell exhaustion. Thus, tryptophan metabolism is best understood as one component of a larger immunometabolic ecosystem in which multiple nutrient axes cooperate to determine whether T cells remain functional, become exhausted, or are excluded from the tumor microenvironment.

## 3 Cellular crosstalk: how tryptophan metabolism builds an immunosuppressive tumor microenvironment

Rather than considering tumor cells, macrophages, CAFs, and lymphocytes as independent targets of tryptophan metabolism, it is more useful to view them as components of a sequential and reciprocal metabolic circuit (40, 78). In this circuit, the key issue is not only which cell type expresses IDO1 or TDO2, but how tryptophan-derived metabolic signals are produced, amplified, spatially organized, and transferred across cellular compartments. Tumor cells may initiate metabolic immune escape, CAFs may stabilize immune-excluded niches, macrophages and MDSCs may amplify suppressive inflammatory states, and lymphocytes represent both direct targets and functional readouts of this network. This perspective shifts the discussion from a pathway-centered model to a multicellular ecosystem model of immune escape. **Table 2** outlines the major cell populations involved in tryptophan-driven tumor immune escape and emphasizes how metabolic crosstalk reshapes the tumor microenvironment at a multicellular level.

**Table 2:** Cellular crosstalk mediated by tryptophan metabolism in the immunosuppressive tumor microenvironment.

Cell type	Tryptophan metabolism-related feature	Effect on other cells	Biological outcome	Representative studies
Tumor cells	Upregulation of IDO1/TDO2 and kynurenine production	Suppress T-cell activity; induce immune tolerance	Immune escape, PD-L1-associated evasion, tumor adaptation	Miyazaki 2022 (57); Shu 2024 (83); Li F 2021 (58); Liang 2022 (65)
Macrophages	Reprogrammed by tryptophan metabolites and inflammatory signaling	Acquire tumor-promoting or M2-like phenotypes	Myeloid immunosuppression and poor immunotherapy response	Xue 2023 (88); Zhao 2021 (89)
Cancer-associated fibroblasts	AHR-dependent stromal signaling; TDO2+ CAF phenotype	Restrict CD8+ T-cell infiltration; reinforce suppressive niche	Immune exclusion, stromal support, resistance	Feng 2022 (78); Lu 2025
Matrix fibroblasts in metastatic niche	TDO2-driven kynurenine signaling	Promote T-cell dysfunction and protect metastatic cells	Lung metastatic outgrowth and niche adaptation	Liu Y 2024 (39)
CD8+ T cells	Exposed to tryptophan depletion and kynurenine accumulation	Reduced proliferation, impaired cytotoxicity, exclusion	Failure of antitumor immunity	Lu 2025; Liu Y 2024 (39); Shu 2024 (83)
Myeloid-enriched suppressive niches	Co-localization with IDO/ARG1 programs	Amplify local immune suppression	Spatially organized immunosuppressive microenvironment	Elomaa 2024 (70)

### 3.1 Tumor cell–intrinsic tryptophan metabolism as a trigger of immune escape

Tumor cells are not merely passive participants in a pre-existing immunosuppressive microenvironment; they actively construct such an environment through metabolic rewiring. One important mechanism by which they do so is the upregulation of tryptophan catabolism. By increasing the expression of IDO1, TDO2, or both, tumor cells establish a metabolic state that serves two functions simultaneously: it supports their own survival and adaptation, and it imposes suppressive constraints on surrounding immune cells. In this sense, tumor cells deploy tryptophan catabolism as both an intrinsic survival program and an extrinsic immunosuppressive cue.

The immune consequences of tumor cell–intrinsic tryptophan metabolism are multifaceted. Enhanced degradation of tryptophan lowers nutrient availability in the local microenvironment, which can weaken lymphocyte proliferation and effector maintenance. At the same time, the resulting accumulation of kynurenine activates AHR-dependent programs that favor immune tolerance and reduce the effectiveness of antitumor responses (79–82). This combination of substrate deprivation and metabolite signaling enables tumor cells to shape the microenvironment in a manner that selectively disadvantages immune surveillance while preserving malignant fitness.

Miyazaki et al. provide one of the clearest examples of this process. In colon cancer, they showed that activation of the TDO2–AHR axis is associated with immune evasion, PD-L1 upregulation, stemness-related features, and liver metastatic potential. This study illustrates how tumor cell–intrinsic tryptophan metabolism can trigger a cascade extending from metabolic remodeling to transcriptional adaptation and immune escape. The significance of this work lies in its demonstration that metabolic and immune phenotypes are tightly coupled: tumor cells do not simply evade immunity and metastasize in parallel, but use the same metabolic axis to support both processes. The tumor-intrinsic dimension of this pathway is further reinforced by Shu et al., who found that PABPC1L induces IDO1 in renal cell carcinoma, thereby promoting immune suppression (83). This study highlights that elevated tryptophan catabolism may be driven by upstream oncogenic or regulatory programs within tumor cells themselves. It also supports the idea that immune escape through tryptophan metabolism is not only a microenvironmental adaptation but also a genetically or post-transcriptionally maintained tumor cell property.

Li et al. extend this concept by showing that TDO2-induced kynurenine signaling in prostate cancer activates AHR/c-Myc-dependent pathways that promote chemotherapy resistance. Although this work is not focused primarily on immune checkpoint biology, it is highly relevant here because it demonstrates how tumor cell–intrinsic tryptophan metabolism can enhance malignant adaptation while reinforcing the broader ecology of immune escape. Tumors that are metabolically primed for survival, plasticity, and stress resistance are also likely to be more capable of withstanding immune-mediated pressure. External environmental cues can further amplify this tumor-intrinsic metabolic program. Liang et al. showed that tobacco carcinogen exposure induces IDO1, thereby enhancing tryptophan metabolism and immune suppression in lung cancer (65). This finding is notable because it suggests that the tumor cell tryptophan-catabolic phenotype can be reinforced not only by intrinsic oncogenic changes but also by carcinogen-driven environmental signaling. Such observations broaden the conceptual scope of the field, indicating that tumor cell–intrinsic immune escape mechanisms may be conditioned by external exposures long before clinical treatment begins.

Together, these studies indicate that tumor cell–intrinsic IDO1 or TDO2 activation can initiate local tryptophan-metabolic immune escape, although its relative importance varies by tumor type and upstream driver.

### 3.2 Tryptophan metabolism and macrophage polarization

Although T-cell dysfunction is often considered the dominant outcome of altered tryptophan metabolism, the myeloid compartment is equally important in determining how this pathway shapes tumor immunity (66, 84). Macrophages, in particular, are highly responsive to metabolic cues within the tumor microenvironment, and growing evidence indicates that tryptophan metabolism can influence their polarization, inflammatory behavior, and tumor-supportive functions (85–87). This is a critical point because it shifts the field away from a lymphocyte-centered view and toward a more integrated model of cellular crosstalk. Macrophage polarization is not a fixed binary process, but tumors frequently exploit signaling pathways that bias macrophages toward immunoregulatory or tumor-promoting phenotypes. In this setting, tryptophan catabolism appears to function as a key instructive signal. Rather than simply suppressing lymphocytes directly, tryptophan metabolism also educates myeloid cells toward immunoregulatory and tumor-supportive states. This effect may occur through direct metabolite signaling, alterations in cytokine networks, or broader reorganization of the local inflammatory niche.

Xue et al. provide important evidence for this concept in breast cancer. Their study showed that tryptophan metabolism regulates inflammatory macrophage polarization and may serve as a predictive factor for immunotherapy response (88). This finding is especially useful for your review because it links mechanistic immunometabolic reprogramming to clinically relevant treatment stratification. It suggests that macrophage states shaped by tryptophan metabolism are not merely descriptive features of the tumor microenvironment, but may influence how tumors respond to immune-based therapy. Zhao et al. further defined this relationship in esophageal squamous cell carcinoma by showing that TDO2 promotes M2 macrophage polarization through an AKT/GSK3β/IL-8 signaling pathway (89). This work is particularly valuable because it connects tryptophan metabolism to a concrete cytokine-associated signaling cascade that actively drives a tumor-promoting macrophage phenotype. The importance of this finding lies in its demonstration that TDO2 does not simply alter metabolite levels in isolation; it remodels the inflammatory context in a way that favors immune suppression and cancer progression.

Support for the broader myeloid relevance of this pathway also comes from tissue-based spatial analysis. Elomaa et al. performed quantitative multiplexed assessment of IDO and ARG1 in colorectal cancer and found associations with myeloid cell infiltration (70). While this study is less focused on mechanistic causality than the others, it contributes an important layer of evidence by showing that amino acid-metabolizing suppressive programs co-localize with myeloid-enriched immunosuppressive niches in human tumors. Such observations strengthen the argument that tryptophan metabolism operates within a broader metabolic architecture that intersects with other suppressive myeloid pathways. From a conceptual standpoint, these findings indicate that tryptophan metabolism can shape the tumor microenvironment indirectly by controlling the functional identity of myeloid cells. This is highly relevant for immune escape, because macrophages influence antigen presentation, T-cell recruitment, cytokine tone, tissue remodeling, and response to immunotherapy. Once reprogrammed toward tumor-supportive states, they can amplify immunosuppression far beyond the direct effects of tryptophan depletion alone. These findings suggest that macrophage reprogramming is an emerging myeloid extension of tryptophan-driven immune escape, but its strength and direction should be interpreted in a tumor-type-specific manner.

### 3.3 Stromal tryptophan metabolism: CAF induction, spatial diffusion, and immune exclusion

Cancer-associated fibroblasts have traditionally been viewed as structural stromal cells responsible for extracellular matrix deposition, tissue stiffening, and support of tumor architecture. However, this view is increasingly incomplete. CAFs are now recognized as metabolically active and immunologically influential components of the tumor microenvironment that can shape cytokine signaling, therapy response, immune cell trafficking, and metastatic niche formation (90–94). In the context of tryptophan metabolism, CAFs should be considered both potential producers and responders within the metabolic network. In some settings, CAFs or matrix fibroblasts may acquire TDO2-positive metabolic phenotypes and directly generate kynurenine-rich signals; in others, tumor-derived cytokines, metabolites, hypoxia, inflammatory cues, or therapy-induced stress may first condition fibroblasts toward an immunosuppressive state. Thus, fibroblast-associated tryptophan metabolism should not be interpreted as a fixed CAF-intrinsic property, but as a context-dependent stromal program shaped by tumor–stroma interactions.

One important conceptual advance is the recognition that CAFs can participate in AHR-centered paracrine signaling networks. Feng et al. showed that CAFs strengthen proliferation and EGFR-TKI resistance in non-small cell lung cancer through AHR-dependent signals (78). Although this study does not establish the entire canonical IDO1/TDO2–Kyn axis in the same direct manner as some tumor-cell-focused reports, it provides an essential foundation for understanding how stromal cells may reinforce tryptophan-linked malignant programs. It supports the idea that the AHR pathway can function as an intercellular relay through which stromal cues amplify tumor-supportive and therapy-resistant states. More direct evidence for a tryptophan-metabolic role of fibroblasts comes from Liu et al., who identified TDO2-positive matrix fibroblasts as important drivers of breast cancer lung metastasis. Their study demonstrated that these fibroblasts promote metastatic outgrowth through kynurenine-mediated ferroptosis resistance in metastatic cells and concomitant T-cell dysfunction. This work is particularly significant because it expands the spatial context of tryptophan metabolism from the primary tumor to the metastatic niche. It also shows that fibroblast-associated tryptophan metabolism can coordinate two hallmarks of metastatic success at once: protection of disseminated cancer cells and suppression of local immune control. Lu et al. provide an even more direct link to immune exclusion. In cutaneous squamous cell carcinoma, they showed that TDO2-positive CAFs mediate immune escape by impeding infiltration of CD8+ T cells (39, 40). Mechanistically, these findings raise several unresolved but important questions. First, kynurenine generated by TDO2-positive CAFs or matrix fibroblasts is unlikely to act only on immediately adjacent cells. Because kynurenine is a diffusible metabolite, local stromal production may generate short-range metabolic gradients that extend from fibroblast-rich regions toward nearby tumor, myeloid, and T-cell compartments. Such gradients may help explain why CD8+ T cells are not merely functionally suppressed but spatially excluded from selected tumor regions. Second, fibroblast-associated tryptophan metabolism may arise through both autonomous and induced mechanisms. CAFs may acquire stable metabolic features during stromal activation, but they may also be induced by tumor-derived cytokines, inflammatory mediators, hypoxia, extracellular matrix remodeling, or therapy-related stress. Third, stromal TDO2 induction is likely to be context-dependent rather than uniform across all CAF states. It may depend on tumor type, tissue site, metastatic organ, local inflammatory tone, AHR-related feedback, and the balance between tumor-derived and immune-derived signals. Fourth, the stability and reversibility of these stromal phenotypes remain insufficiently defined. The observation that TDO2 inhibition can increase CD8+ T-cell infiltration in selected models suggests that at least part of fibroblast-associated immune exclusion may be pharmacologically reversible, but whether this reflects direct CAF reprogramming, altered metabolite gradients, or secondary immune remodeling remains unclear. This finding is especially important for the current review because it moves beyond the general concept of stromal immunosuppression and identifies a specific fibroblast-associated metabolic program that shapes the spatial accessibility of antitumor lymphocytes. In other words, tryptophan metabolism in CAFs may not only weaken T-cell function, but may also physically and functionally organize CD8-low or CD8-excluded tumor regions. These studies together support a more advanced view of CAF biology in tumor immune escape. CAFs should not be considered merely matrix-producing support cells, but rather metabolic and immunological organizers that can intensify tryptophan-driven suppression through paracrine signaling, niche conditioning, and control of immune-cell access. Their influence is likely to be especially important in tumors characterized by strong stromal remodeling, therapy resistance, or metastatic tropism. This fibroblast-centered perspective also has important implications for therapeutic design. If tryptophan metabolism is partly sustained or amplified by stromal populations, targeting tumor cells alone may not be sufficient to reverse immune suppression. Effective intervention may require disruption of the intercellular metabolic circuitry that links tumor cells, CAFs, and immune populations. For this reason, CAF-mediated tryptophan signaling should be viewed as an emerging stromal mechanism that requires spatial and functional validation. Future studies should determine whether TDO2-positive CAFs represent a stable fibroblast subset, a transient activation state, or a reversible metabolic phenotype induced by tumor-derived and inflammatory cues. Integrating spatial transcriptomics, multiplex imaging, and spatial metabolomics will be essential for mapping TDO2-expressing fibroblasts, kynurenine-enriched regions, myeloid infiltration, and CD8+ T-cell exclusion within the same tissue architecture.

### 3.4 Spatial organization of stromal tryptophan-metabolic niches

The stromal dimension of tryptophan metabolism is intrinsically spatial (95, 96). Bulk transcriptomic or metabolomic approaches can identify IDO1, TDO2, AHR-related programs, or kynurenine-pathway activity, but they cannot determine whether these signals arise from tumor cells, CAFs, myeloid cells, or spatially restricted interface regions (97, 98). This distinction is critical because a tryptophan-metabolic niche may function through local gradients rather than uniform tumor-wide activity. For example, kynurenine produced by TDO2-positive CAFs or matrix fibroblasts may diffuse across short distances and affect neighboring CD8+ T cells, macrophages, dendritic cells, or metastatic tumor cells (39, 40). In this model, immune escape is not simply caused by global tryptophan depletion, but by spatially organized metabolite exposure at tumor–stroma or metastatic niche interfaces.

Spatial transcriptomics can help define where TDO2-positive CAFs, IDO1-high myeloid cells, AHR-responsive tumor cells, and excluded CD8+ T cells are located relative to one another (95). Multiplex imaging can further determine whether these populations physically co-localize in fibroblast-rich or myeloid-enriched suppressive niches (70, 95). Spatial metabolomics provides an additional layer by mapping kynurenine, tryptophan, indole-related metabolites, or NAD^+^-linked metabolic products within intact tissue sections (99, 100). Integrating these technologies would allow future studies to test several unresolved mechanisms: whether stromal TDO2 expression is induced at tumor–stroma boundaries, whether kynurenine gradients overlap with CD8-low regions, whether CAF-associated tryptophan metabolism is stable or reversible after pathway inhibition, and whether metastatic niches contain distinct tryptophan-metabolic architectures compared with primary tumors (40, 97, 101). This spatial perspective also has therapeutic implications. If stromal tryptophan metabolism is organized into localized suppressive niches, then bulk IDO1/TDO2 expression or circulating kynurenine/tryptophan ratios may be insufficient for patient selection. Instead, clinically meaningful biomarkers may need to integrate enzyme expression, metabolite distribution, stromal identity, myeloid localization, and T-cell accessibility (96, 101). Such spatially resolved profiling could identify tumors in which CAF- or myeloid-associated tryptophan metabolism is a true driver of immune exclusion rather than a secondary feature of an already immunosuppressed tumor microenvironment.

### 3.5 CD8+ T-cell dysfunction and immune exclusion

The spatial organization of tryptophan-metabolic niches ultimately converges on a central immunological consequence: impaired CD8+ T-cell access and function. In this context, CD8+ T-cell dysfunction should be considered not only as a cell-intrinsic exhaustion or suppression state, but also as a spatial outcome shaped by metabolite gradients, CAF-rich barriers, myeloid-enriched niches, and altered tumor–stroma interfaces. Cytotoxic T lymphocytes are among the most important effectors of immune control in cancer, and their functional integrity depends on adequate nutrient access, effective activation signals, and permissive tissue localization (102–106). Tryptophan metabolism undermines each of these requirements. At the most basic level, local tryptophan depletion compromises T-cell proliferation and effector maintenance by imposing metabolic stress within the tumor microenvironment. This nutrient restriction is compounded by kynurenine accumulation, which further promotes dysfunctional immune states through receptor-mediated signaling, especially via AHR. As a result, tryptophan metabolism exerts a dual pressure on CD8+ T cells: it deprives them of a critical substrate while simultaneously exposing them to suppressive metabolites that blunt their cytotoxic competence. Recent studies indicate that the impact of this pathway extends beyond functional impairment to spatial exclusion. Lu et al. showed that TDO2-positive CAFs impede CD8+ T-cell infiltration in cutaneous squamous cell carcinoma, providing a clear example of how tryptophan metabolism can shape not only lymphocyte activity but also lymphocyte access to the tumor bed. This is an important distinction. In some tumors, immune escape may not depend solely on generating exhausted or suppressed T cells within the lesion, but on preventing their effective entry into key tumor regions in the first place.

Liu et al. reinforce this view in the metastatic setting. In breast cancer lung metastasis, TDO2-positive matrix fibroblasts promoted T-cell dysfunction while simultaneously supporting survival of metastatic cells. Their findings suggest that the tryptophan-metabolic niche can protect metastatic lesions not only by fortifying tumor cells against ferroptotic stress, but also by weakening the immune cells that would otherwise eliminate disseminated disease. This coordination of immune dysfunction and metastatic fitness may be especially relevant in organs where stromal remodeling plays a dominant role in colonization and outgrowth. Tumor-cell-intrinsic studies also point to direct links between tryptophan metabolism and immune checkpoint-associated escape. Miyazaki et al. showed that the TDO2–AHR pathway in colon cancer is associated with PD-L1-mediated immune evasion, indicating that tryptophan catabolism may intersect with established checkpoint circuits. Similarly, Shu et al. demonstrated in renal cell carcinoma that IDO1-associated programs are linked to impaired T-cell function, further emphasizing that tumors can use tryptophan metabolism to weaken cytotoxic immunity through both metabolic and immunoregulatory routes.

**Figure 2:**
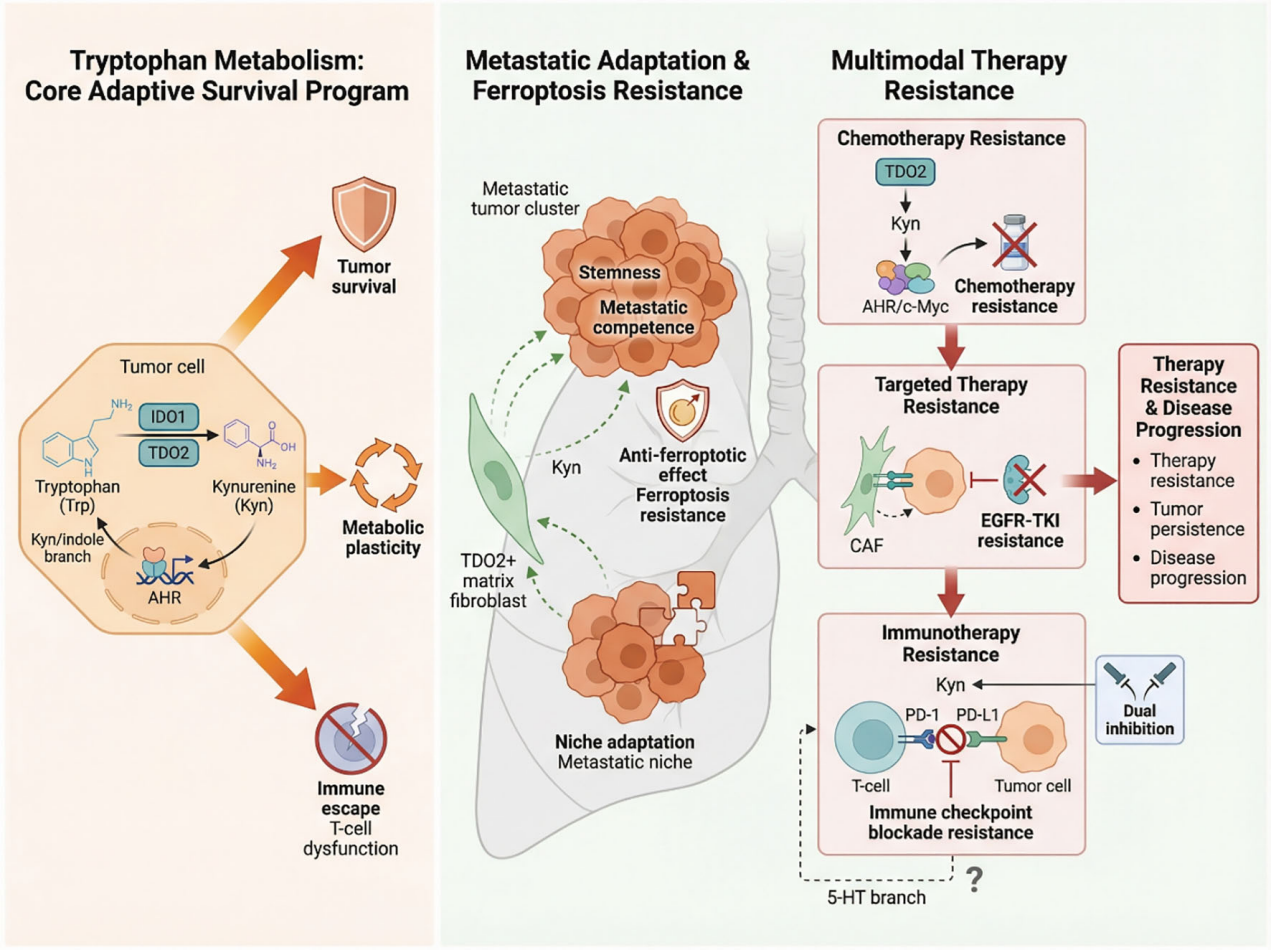
Tryptophan metabolism drives multicellular crosstalk in the immunosuppressive tumor microenvironment. Tumor cell–intrinsic IDO1/TDO2 activity depletes tryptophan and increases kynurenine, thereby promoting AHR-dependent immune evasion. This metabolic program reprograms macrophages, activates CAF-mediated stromal support, impairs CD8+ T-cell function, and drives immune exclusion, metastatic adaptation, and therapy resistance. This image was created using Adobe Illustrator.

Taken together, these findings support a model in which tryptophan metabolism drives a spectrum of CD8+ T-cell impairment ranging from proliferative and functional suppression to spatial exclusion from the tumor microenvironment. This is a particularly powerful feature of the pathway because it means that tryptophan metabolism can affect both the quality and the localization of antitumor immune responses. As such, it represents a central mechanism through which tumors convert a metabolically altered microenvironment into a durable state of immune escape. **Figure 2** illustrates how tryptophan metabolism extends beyond tumor cells to coordinate macrophages, CAFs, and CD8+ T cells within an immunosuppressive tumor microenvironment. It highlights that tumor immune escape is sustained by multicellular metabolic crosstalk rather than by a tumor-cell-intrinsic pathway alone.

### 3.6 Additional immune populations involved in tryptophan metabolism-driven immune escape

Beyond macrophages and CD8+ T cells, additional immune populations also contribute to tryptophan metabolism-driven immune escape (18, 107). Regulatory T cells (Tregs) can be expanded and functionally reinforced by kynurenine–AHR signaling, thereby promoting local immune tolerance and suppressing effector T-cell responses (41, 42). Myeloid-derived suppressor cells (MDSCs) may also be recruited or activated in response to tryptophan catabolism, further inhibiting cytotoxic T-cell function and strengthening the immunosuppressive myeloid compartment (107). Natural killer (NK) cells can be impaired by tryptophan depletion and kynurenine accumulation, leading to reduced cytotoxic activity against tumor cells (43, 108). Dendritic cells may also acquire tolerogenic features under IDO1-high or kynurenine-rich conditions, resulting in defective antigen presentation and enhanced T-cell suppression (107, 109). Together, these immune populations interact with tumor cells, macrophages, and stromal elements to form a broader immunosuppressive network that extends beyond CD8+ T cells and macrophages.

## 4 Tryptophan metabolism beyond immune suppression: tumor adaptation, metastasis, and therapy resistance

Beyond its immunological effects, emerging evidence links tryptophan metabolism to tumor-intrinsic adaptation, including ferroptosis resistance, stemness-like phenotypes, metastatic niche formation, and therapy resistance. Because these mechanisms are newer and more context-dependent than the canonical T-cell suppression axis, they are discussed here as emerging extensions rather than universally established outputs.

### 4.1 Tryptophan metabolism supports tumor survival and ferroptosis resistance

One of the most important recent advances in the field is the recognition that tryptophan metabolism can directly promote tumor cell survival by suppressing ferroptosis (110–112). Ferroptosis is a regulated form of cell death driven by iron-dependent lipid peroxidation, and it has emerged as a key vulnerability in multiple cancers. Because ferroptotic sensitivity is strongly influenced by metabolic state, pathways that enhance redox balance or limit lipid peroxidation can substantially improve tumor cell fitness. This relationship also provides an example of why tryptophan metabolism should not be reduced to Kyn–AHR signaling alone. Downstream kynurenine-pathway metabolites can influence oxidative stress handling, mitochondrial metabolism, and NAD^+^-dependent adaptive responses, all of which may affect ferroptotic vulnerability. Quinolinic acid, for example, connects tryptophan catabolism to *de novo* NAD^+^ biosynthesis and has been reported to confer oxidative stress resistance in glioma. Therefore, the anti-ferroptotic effects of tryptophan metabolism may involve broader redox and bioenergetic rewiring in addition to AHR-dependent transcriptional programs. In this context, tryptophan metabolism appears to function as an adaptive survival program.

Liu et al. provided strong evidence for this concept by demonstrating that tryptophan metabolism acts as a previously underappreciated anti-ferroptotic pathway that supports tumor growth (113). This finding is highly significant because it broadens the biological role of tryptophan metabolism beyond immune regulation and places it within the larger framework of stress adaptation and metabolic resilience. It suggests that tumors may exploit tryptophan catabolism not only to suppress immune attack but also to protect themselves from oxidative damage and ferroptotic collapse (114–116). Such a function would be particularly advantageous in metabolically unstable and treatment-exposed tumor environments. This anti-ferroptotic role becomes even more compelling in the metastatic context. Liu et al. later showed that TDO2-positive matrix fibroblasts in breast cancer lung metastasis promote kynurenine-mediated ferroptosis resistance in metastatic cells while simultaneously impairing T-cell function. This study is especially important because it links stromal tryptophan metabolism to metastatic outgrowth and shows that survival signaling and immune suppression are tightly coupled in the metastatic niche. Rather than acting independently, these two processes appear to reinforce each other: protection from ferroptosis helps disseminated tumor cells persist, while immune dysfunction reduces the likelihood of their elimination. This convergence has important conceptual implications. If tryptophan metabolism supports both ferroptosis resistance and immune escape, then tumors gain a dual survival advantage from activating the same pathway. They become less susceptible to both cell-intrinsic death signals and cell-extrinsic immune pressure. As a result, the anti-ferroptotic function of tryptophan metabolism should not be viewed as a separate phenomenon from immune escape, but as part of a broader adaptive strategy through which tumors stabilize survival under hostile conditions.

Taken together, these findings indicate that tryptophan metabolism contributes directly to tumor persistence by reinforcing metabolic defenses against ferroptotic stress. This survival benefit is likely to cooperate with immunosuppressive signaling, thereby enabling tumors to withstand both endogenous stress and therapeutic challenge more effectively.

### 4.2 Tryptophan metabolism promotes stemness, metastatic competence, and niche adaptation

Beyond supporting immediate survival, tryptophan metabolism also appears to facilitate longer-term malignant adaptation by promoting stemness, metastatic competence, and successful niche colonization (117–119). These functions are highly relevant to tumor progression because metastatic dissemination and recurrence require more than immune evasion alone; they depend on the ability of tumor cells to remain plastic, survive in foreign microenvironments, and exploit supportive stromal interactions (120, 121). Miyazaki et al. provided one of the clearest demonstrations of this principle by showing that the TDO2–AHR pathway is associated with stemness and immune evasion in colon cancer liver metastasis. Their findings indicate that tryptophan metabolism can connect transcriptional programs related to self-renewal and metastatic behavior with immune checkpoint-associated escape. This is particularly important because it suggests that stem-like traits and immune resistance may not arise independently but may instead be co-organized by the same metabolic axis.

The role of tryptophan metabolism in niche adaptation is further highlighted by the study of Liu et al., which identified TDO2-positive matrix fibroblasts as facilitators of breast cancer lung metastasis. In this context, fibroblast-derived kynurenine-mediated signaling helped establish a supportive metastatic niche by protecting disseminated tumor cells from ferroptotic injury and weakening local T-cell surveillance. This work expands the importance of tryptophan metabolism from the primary tumor microenvironment to the ecology of metastatic seeding and outgrowth. It suggests that successful metastasis depends not only on tumor-cell-intrinsic metabolic programs but also on stromal support systems that recreate immunosuppressive and survival-promoting conditions at distant sites. Li et al. add another layer to this adaptive model by showing that TDO2-driven kynurenine signaling activates AHR/c-Myc-associated programs in prostate cancer, thereby promoting chemotherapy resistance. Although their primary focus was treatment response, the broader implication is that tryptophan metabolism contributes to malignant plasticity and adaptation under therapeutic stress. Tumor cells able to activate this axis may be better equipped to survive in changing microenvironments, resist hostile stimuli, and preserve proliferative competence. Similarly, Feng et al. showed that CAF-associated AHR-dependent signaling enhances proliferation and EGFR-TKI resistance in non-small cell lung cancer. This study supports the idea that stromal tryptophan-linked signaling pathways can reinforce adaptive tumor states, particularly in settings where therapy imposes selective pressure. The significance of this work lies in its demonstration that metabolic niche adaptation is not solely a tumor-cell-autonomous process, but rather a cooperative event involving tumor cells and stromal populations.

Together, these studies suggest that tryptophan metabolism contributes to a broader metastatic and adaptive phenotype characterized by stemness, survival plasticity, stromal cooperation, and niche conditioning. Such functions elevate the pathway from a mechanism of immune suppression to a central regulator of malignant progression.

### 4.3 Tryptophan metabolism as a driver of treatment resistance

The contribution of tryptophan metabolism to treatment resistance is one of the most clinically relevant aspects of this field. If this pathway merely accompanied immune suppression, its therapeutic importance would remain limited. However, accumulating evidence indicates that tryptophan metabolism actively drives resistance across multiple treatment modalities, including chemotherapy, targeted therapy, and immune checkpoint blockade (122–126). This reinforces the notion that it is a functional axis of tumor persistence rather than a passive biomarker of advanced disease. Chemotherapy resistance is exemplified by the work of Li et al., who showed that TDO2-induced kynurenine signaling promotes chemoresistance in prostate cancer through an AHR/c-Myc-dependent mechanism. This study suggests that altered tryptophan metabolism can support tumor survival under cytotoxic stress by activating transcriptional programs that preserve malignant fitness. Importantly, such tumor-intrinsic resistance mechanisms may coexist with immune escape, thereby making treatment failure more difficult to reverse.

A related phenomenon is observed in targeted therapy resistance. Feng et al. demonstrated that cancer-associated fibroblasts enhance EGFR-TKI resistance in non-small cell lung cancer through AHR-dependent signaling. Although this study is framed in the context of stromal support and AHR activity rather than classical enzymatic tryptophan depletion, it nonetheless highlights how tryptophan-linked signaling circuits can reinforce tumor adaptation to targeted agents. It also emphasizes that resistance may emerge from tumor–stromal cooperation rather than exclusively from tumor-cell-autonomous genetic changes. The relationship between tryptophan metabolism and resistance is especially striking in immunotherapy (127, 128). Oldan et al. reported that increased tryptophan metabolism, but not increased glucose metabolism, predicts resistance to pembrolizumab in advanced melanoma. This observation is particularly important because it supports the idea that tryptophan catabolism may serve as a more relevant metabolic indicator of immune checkpoint blockade failure than broader measures of metabolic activity. Likewise, Liang et al. showed that tobacco carcinogen-induced IDO1 upregulation promotes immune suppression and is associated with poorer anti-PD-1 responsiveness, further linking environmental exposure, metabolic reprogramming, and immunotherapy outcome.

**Figure 3:**
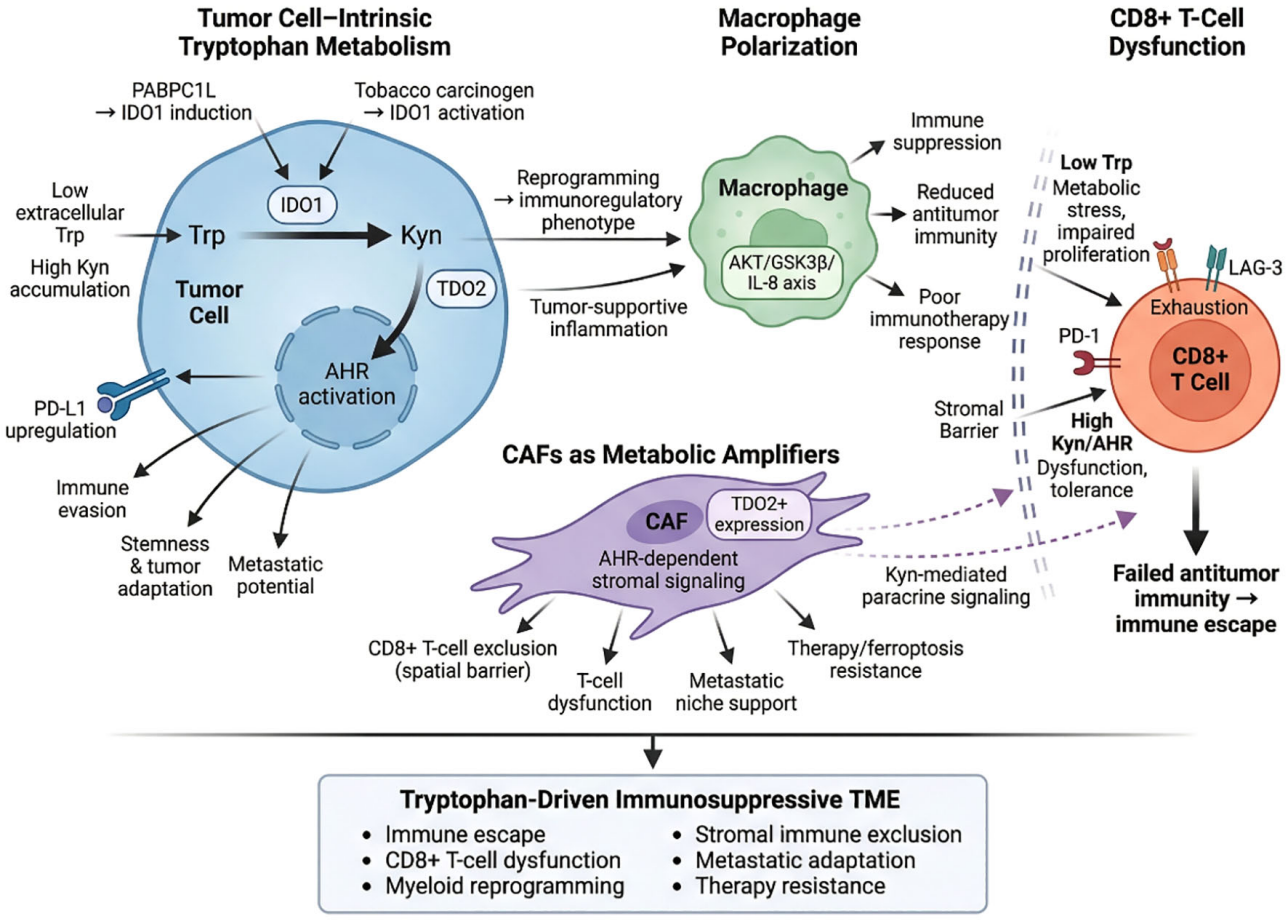
Tryptophan metabolism promotes tumor adaptation, metastasis, and therapy resistance beyond immune suppression. The Trp–IDO1/TDO2–Kyn–AHR axis supports tumor survival by enhancing ferroptosis resistance, stemness, metastatic niche adaptation, and multicellular stromal support. This adaptive network also contributes to resistance to chemotherapy, targeted therapy, and immune checkpoint blockade through branched metabolic signaling and tumor–stromal cooperation. This image was created using Adobe Illustrator.

Additional support comes from Li et al., who demonstrated in ovarian cancer that rewiring tryptophan metabolism through dual inhibition of the Kyn/indole and 5-HT branches can restore sensitivity to immune checkpoint blockade. This work is particularly instructive because it suggests that therapy resistance may be sustained not only by one canonical enzymatic node but by a broader metabolic network. In this context, effective reversal of resistance may require coordinated intervention across multiple branches of tryptophan metabolism rather than selective blockade of a single enzyme. Wu et al. extend this therapeutic logic in platinum-resistant non-small cell lung cancer by showing that dual inhibition of IDO1 and TDO2 enhances antitumor immunity more effectively than single-target approaches. Their findings highlight the possibility of compensatory pathway usage and metabolic redundancy in resistant tumors. This is a critical message for the field: tumors may preserve immunosuppressive and survival outputs through overlapping tryptophan-catabolic routes, thereby limiting the efficacy of monotherapies. Overall, the available evidence strongly supports the conclusion that tryptophan metabolism is not a mere bystander during treatment failure. Instead, it acts as a driver of resistance by coordinating immune suppression, tumor-intrinsic adaptation, stromal support, and metabolic plasticity. Recognizing this role is essential for moving the field beyond descriptive immunometabolism and toward mechanistically informed therapeutic strategies. **Figure 3** illustrates that tryptophan metabolism functions not only as an immunosuppressive pathway but also as a broader adaptive program that promotes tumor persistence under therapeutic and microenvironmental stress. It highlights how ferroptosis resistance, metastatic niche support, and branched metabolic signaling converge to drive malignant progression and treatment failure.

### 4.4 Tumor cell-intrinsic and non-enzymatic IDO1 functions complicate therapeutic targeting

An emerging layer of complexity is that IDO1 may promote tumor progression not only through its canonical catalytic activity, but also through tumor cell-intrinsic and non-enzymatic signaling functions (44, 45). Traditionally, IDO1 has been interpreted mainly as an immunosuppressive enzyme that depletes tryptophan and generates kynurenine, thereby suppressing effector T-cell activity and promoting immune tolerance (18). However, recent studies suggest that this enzyme-centric model is incomplete (44). IDO1 can also act as a signaling scaffold in selected cellular contexts, and this non-enzymatic activity may support malignant proliferation, migration, and adaptation independently of kynurenine production (45). This concept is particularly important for understanding the limited efficacy of catalytic IDO1 inhibition. Epacadostat and related inhibitors were developed to block IDO1 enzymatic activity and reduce kynurenine-mediated immune suppression. Nevertheless, recent evidence indicates that catalytic inhibition may not necessarily eliminate all tumor-promoting functions of IDO1 (44, 45). Instead, some catalytic inhibitors may stabilize non-enzymatic conformations of IDO1 that remain capable of intracellular signal transduction. In IDO1-expressing tumor cells, this effect may enhance pro-tumorigenic signaling and thereby counteract the expected therapeutic benefit of enzymatic blockade (45). Thus, the biological output of IDO1 inhibition may depend not only on the reduction of kynurenine production, but also on how inhibitor-bound IDO1 influences non-catalytic protein functions within tumor cells (44). This tumor cell-intrinsic activity helps explain why tryptophan metabolism should not be viewed solely as an extracellular immunosuppressive pathway. Rather, IDO1 may function as a moonlighting protein that links metabolic enzyme activity with intracellular oncogenic signaling. This duality has major implications for therapeutic design: strategies that only inhibit catalytic activity may be insufficient, or in some contexts even biologically unfavorable, if the IDO1 protein remains stabilized and signal-competent. Therefore, future studies should distinguish between catalytic, non-catalytic, immune-cell-dependent, and tumor cell-intrinsic functions of IDO1 when evaluating tryptophan metabolism-targeted therapies.

## 5 Clinical translation: biomarkers and patient stratification

The translational relevance of tryptophan metabolism in cancer lies not only in its mechanistic importance but also in its potential utility for biomarker development and patient stratification. Because this pathway integrates metabolic remodeling with immune dysfunction, it may offer clinically informative readouts that reflect both tumor biology and microenvironmental state. This is particularly valuable in the era of precision oncology, where identifying patients with immunosuppressive metabolic phenotypes may improve therapeutic selection, monitoring, and combination design.

### 5.1 Circulating tryptophan metabolites as blood-based biomarkers

One of the most appealing translational applications of tryptophan metabolism is the use of circulating metabolites as minimally invasive biomarkers. Blood-based measures have several practical advantages, including repeatability, accessibility, and potential utility in real-time monitoring (129–131). Among these, the kynurenine/tryptophan ratio has emerged as one of the most commonly studied indicators of systemic tryptophan catabolism. It should be noted that the reliability of blood-based biomarkers can be influenced by various environmental and external factors, including diet, circadian rhythm, comorbidities, and sample handling. This limitation should be considered when interpreting biomarker measurements and their potential clinical utility.

Mandarano et al. showed that the kynurenine/tryptophan ratio may serve as a potential blood-based biomarker in non-small cell lung cancer (132). Although such biomarkers do not fully capture local intratumoral metabolism, they provide an important starting point for translating immunometabolic knowledge into clinically usable assays. Their potential value lies in reflecting the net balance between substrate consumption and metabolite generation, which may correspond to an immunosuppressive metabolic state. Oldan et al. further highlight the clinical significance of this concept by showing that increased tryptophan metabolism predicts pembrolizumab resistance in advanced melanoma. This finding suggests that metabolic activity along the tryptophan pathway may have prognostic and predictive value in the immunotherapy setting. Importantly, it also raises the possibility that circulating or imaging-linked metabolic indicators could help identify patients less likely to benefit from immune checkpoint blockade alone. Taken together, these studies suggest that tryptophan-related metabolites have promise as noninvasive biomarkers for patient stratification and therapeutic monitoring. While further validation is needed, especially across cancer types and treatment settings, the ability to repeatedly assess an immunometabolic axis through blood-based approaches represents a major translational opportunity.

### 5.2 Spatial and tissue-level metabolic immune profiling

While circulating biomarkers are attractive for their practicality, tissue-level analysis remains essential for understanding how tryptophan metabolism is organized within tumors. A major challenge in clinical translation is that immunosuppressive metabolism is often spatially heterogeneous. Local expression of IDO1, TDO2, AHR-related programs, and accompanying immune-cell infiltration may vary substantially across tumor regions, metastatic sites, and stromal compartments. Accordingly, tissue-based profiling provides a more precise view of where and how tryptophan metabolism contributes to immune escape. Elomaa et al. offer an important example through their multiplexed spatial analysis of IDO, ARG1, and myeloid infiltration in colorectal cancer. Their work suggests that amino acid-metabolizing suppressive pathways are embedded within distinct tissue niches rather than being uniformly distributed. This is a key point for clinical interpretation: the biological meaning of tryptophan metabolism may depend not only on how much pathway activity is present, but also on where that activity is localized relative to immune and stromal populations (133–135). Shu et al. add mechanistic depth to tissue-level profiling by showing that IDO1 induction in renal cell carcinoma is associated with immune suppression and impaired T-cell function. Their study illustrates how tissue expression of pathway regulators can reveal immune escape programs that may not be fully apparent from systemic measurements alone. Similarly, Miyazaki et al. provide a tissue-context example in which the TDO2–AHR axis is linked to PD-L1 expression, stemness, and metastasis in colon cancer, showing that spatially localized metabolic programs may integrate immune and malignant phenotypes within the same lesion. These observations support the growing importance of spatial and tissue-resolved metabolic immune profiling in oncology. Rather than treating tryptophan metabolism as a uniform tumor-wide variable, future clinical strategies may need to identify specific metabolic niches characterized by stromal support, myeloid enrichment, checkpoint activation, or immune exclusion. Such tissue-based profiling could improve biomarker interpretation and help guide combination therapy.

### 5.3 Tryptophan metabolism for immunotherapy response prediction

Among the translational applications of tryptophan metabolism, prediction of immunotherapy response is perhaps the most clinically compelling (136, 137). Because immune checkpoint blockade depends on the presence of functionally competent antitumor immunity, metabolic programs that suppress T-cell activity, reprogram myeloid cells, or generate immune-excluded niches are highly likely to influence treatment outcome.

Oldan et al. provide direct support for this concept by showing that increased tryptophan metabolism predicts resistance to pembrolizumab in melanoma (138). This study implies that assessment of tryptophan-related metabolic activity may improve identification of patients unlikely to derive durable benefit from anti-PD-1 monotherapy. Xue et al. complement this perspective by demonstrating that tryptophan metabolism regulates inflammatory macrophage polarization and may serve as a predictive factor for breast cancer immunotherapy. Their findings broaden the predictive relevance of this pathway beyond T-cell-centered mechanisms and suggest that myeloid reprogramming may be an important component of immunotherapy resistance. Liang et al. add an exposure-linked dimension by showing that tobacco carcinogen-induced IDO1 activation contributes to immune suppression and inferior anti-PD-1 responsiveness. This suggests that environmental factors capable of stimulating tryptophan metabolism may indirectly shape immunotherapy outcome. Meanwhile, Li et al. provide proof-of-concept that rewiring branched tryptophan metabolism can restore checkpoint blockade sensitivity in ovarian cancer, implying that the same pathway may serve both as a predictive marker and a therapeutic target in resistant disease.

Together, these studies indicate that tryptophan metabolism has substantial potential as a framework for immunotherapy response prediction. Its value likely lies in reflecting an integrated state of immune suppression, stromal conditioning, and tumor adaptation rather than a single isolated variable. As clinical immunotherapy becomes increasingly personalized, metabolic profiling of the tryptophan axis may help define biologically meaningful subgroups of patients with distinct therapeutic vulnerabilities.

## 6 Therapeutic opportunities: how to target tryptophan metabolism in cancer

The network-based interpretation of tryptophan metabolism fundamentally changes therapeutic strategy. In an enzyme-centered model, the logical intervention is to inhibit IDO1 or TDO2 and thereby reduce kynurenine-mediated immune suppression. In a multicellular network model, however, therapeutic failure may arise from several non-mutually exclusive mechanisms: compensatory IDO1/TDO2 activity, metabolic branching into non-kynurenine routes, stromal or myeloid maintenance of suppressive niches, spatial exclusion of CD8+ T cells, and non-enzymatic IDO1 signaling. Therefore, effective intervention should not be limited to catalytic inhibition of a single enzyme. Instead, future strategies should combine pathway blockade with patient stratification, spatial metabolic profiling, immune checkpoint blockade, myeloid or CAF reprogramming, and approaches capable of targeting both enzymatic and non-enzymatic functions of IDO1.

### 6.1 Why single-node inhibition is often insufficient

A major challenge in targeting tryptophan metabolism is that tumors may not rely on a single catabolic enzyme or a single downstream metabolite. Functional redundancy between IDO1 and TDO2, as well as branching into multiple metabolic routes, can allow tumors to preserve immunosuppressive outputs even when one node is blocked. Wu et al. provide strong evidence for this issue by showing that dual inhibition of IDO1 and TDO2 enhances antitumor immunity in platinum-resistant non-small cell lung cancer more effectively than single-target inhibition. Their findings suggest that tumors may compensate for blockade of one enzyme by maintaining flux through the other, thereby sustaining kynurenine production and immune escape. This has important therapeutic implications, particularly in resistant tumors that have already evolved robust adaptive programs. Li et al. extend the problem beyond enzymatic redundancy by showing that tryptophan metabolism is branched rather than strictly linear. Their work in ovarian cancer indicates that targeting only the canonical kynurenine route may be insufficient if parallel branches such as indole- or 5-HT-related metabolism continue to support tumor survival and immunotherapy resistance. Together, these studies argue that focusing narrowly on a single metabolic node may fail to remodel the broader immunosuppressive network. These findings suggest that rational combinations should be selected according to the dominant resistance mechanism in each tumor context, such as IDO1/TDO2 redundancy, branched metabolic flux, stromal niche protection, or persistent non-enzymatic IDO1 signaling.

A further therapeutic challenge is that catalytic inhibition may not fully neutralize IDO1 biology. Recent findings indicate that IDO1 has non-enzymatic signaling activity in addition to its canonical tryptophan-catabolic function. In this context, conventional catalytic inhibitors may suppress kynurenine production but leave the IDO1 protein intact, allowing non-catalytic signaling to persist. More importantly, some inhibitors may stabilize a non-enzymatic IDO1 conformation that promotes tumor cell-intrinsic signaling, proliferation, and migration. This possibility provides a mechanistic explanation for why catalytic IDO1 blockade may fail to generate durable antitumor benefit in some settings. Therefore, future therapeutic development should move beyond simple enzymatic inhibition. More comprehensive approaches may include agents capable of blocking both catalytic and non-catalytic functions of IDO1, disrupting IDO1-associated signaling partners, or inducing IDO1 protein degradation. Such strategies may be particularly relevant in tumors with high tumor cell-intrinsic IDO1 expression, where the protein itself may contribute to malignant behavior independently of kynurenine production. Incorporating this dual-function model into trial design may improve patient selection and help avoid therapeutic strategies that reduce enzymatic activity while unintentionally preserving or enhancing IDO1-dependent pro-tumorigenic signaling.

### 6.2 Lessons from failed IDO1-targeted clinical trials

The failure of IDO1-targeted therapy in late-stage clinical development provides one of the most important translational lessons in cancer immunometabolism. Epacadostat, a selective IDO1 enzymatic inhibitor, showed encouraging activity in early-phase studies when combined with pembrolizumab, supporting the hypothesis that blocking IDO1-mediated tryptophan catabolism could enhance immune checkpoint blockade (139). However, this rationale was not confirmed in the phase III ECHO-301/KEYNOTE-252 trial, in which epacadostat plus pembrolizumab failed to improve progression-free survival or overall survival compared with pembrolizumab alone in patients with unresectable or metastatic melanoma (140). This discrepancy between preclinical rationale, early clinical signals, and phase III failure suggests that IDO1 inhibition cannot be interpreted as a universally effective strategy for reversing tumor immune escape.

Several explanations should be considered. First, pharmacodynamic inhibition may have been insufficient. Although epacadostat was administered at 100 mg twice daily in ECHO-301, subsequent analyses raised the possibility that this dose may not have produced sustained suppression of systemic kynurenine production in all patients. Second, patient selection was not based on a validated tryptophan-metabolic biomarker. Patients were not prospectively enriched for IDO1-high tumors, high kynurenine/tryptophan ratios, AHR-active tumors, or spatially defined IDO1-positive suppressive niches (141, 142). Third, IDO1 may not be the dominant driver of immunosuppressive tryptophan metabolism in all tumors. TDO2, IDO2, IL4I1, microbial indole pathways, and 5-HT-associated branches may preserve immunosuppressive or tumor-supportive outputs even when IDO1 is inhibited (37). Fourth, kynurenine may not be the only relevant suppressive metabolite. Alternative tryptophan-derived metabolites can also activate AHR-dependent or AHR-independent programs, suggesting that a kynurenine-centered biomarker may incompletely represent pathway activity. Fifth, the relevant source of tryptophan metabolism may not always be tumor-cell IDO1. Stromal fibroblasts, macrophages, dendritic cells, MDSCs, and other non-malignant compartments can generate or amplify immunosuppressive metabolic niches, meaning that tumor-cell enzyme expression alone may be an inadequate therapeutic guide. Finally, emerging evidence indicates that IDO1 can exert non-enzymatic, tumor cell-intrinsic signaling functions; therefore, catalytic inhibition may reduce kynurenine production without fully eliminating IDO1-dependent tumor-promoting activity.

Other agents further illustrate the complexity of this field. Indoximod differs from direct enzymatic IDO1 inhibitors because it acts as a tryptophan mimetic and may modulate immune-cell responses downstream of IDO activity (142, 143). Clinical studies have shown acceptable tolerability and potential activity in selected settings, but randomized biomarker-driven validation remains limited (141, 142, 144). Linrodostat is a potent oral IDO1 inhibitor with pharmacodynamic evidence of kynurenine suppression, yet early clinical data suggest that kynurenine reduction alone may not reliably predict clinical response (145, 146). Dual IDO1/TDO2 inhibitors may better address enzymatic redundancy, and AHR inhibitors may bypass upstream enzyme compensation by targeting a convergent downstream signaling node (35, 37, 147). However, these approaches also require careful biomarker development because AHR biology is context-dependent and may vary across tumor types, immune compartments, and stromal states (41, 147).

The main lesson from IDO1-targeted clinical failure is therefore not that tryptophan metabolism is therapeutically irrelevant, but that it cannot be targeted effectively as a single, uniform, enzyme-centered pathway. Future trials should incorporate pharmacodynamic confirmation of pathway inhibition, baseline and on-treatment kynurenine/tryptophan assessment, tissue-level IDO1/TDO2/IL4I1 profiling, AHR activity signatures, spatial analysis of stromal and myeloid niches, and rational combinations with immune checkpoint blockade or myeloid/CAF-directed therapies. Such strategies may help identify the subset of tumors in which tryptophan metabolism is a true driver of immune escape rather than a correlative marker of an already immunosuppressed microenvironment.

### 6.3 Combination strategies with immunotherapy

Because tryptophan metabolism directly impairs antitumor immunity, one of the most logical therapeutic directions is to combine metabolic intervention with immune checkpoint blockade (148–150). The rationale is straightforward: metabolic rewiring may reduce the suppressive constraints that prevent reinvigoration of T cells, while anti-PD-1 or anti-PD-L1 therapy can then restore effector activity more effectively. Wu et al. support this concept by demonstrating that dual targeting of IDO1 and TDO2 enhances antitumor immune responses in resistant non-small cell lung cancer. Liang et al. add translational relevance by showing that tobacco carcinogen-induced IDO1 activation contributes to poor anti-PD-1 response, implying that patients with high tryptophan-catabolic activity may benefit from combined metabolic and checkpoint-directed intervention. Oldan et al. further reinforce this rationale by showing that increased tryptophan metabolism predicts pembrolizumab resistance, suggesting that pathway activity itself may identify candidates for combination approaches. Li et al. offer an especially forward-looking model by demonstrating that rewiring branched tryptophan metabolism can restore checkpoint blockade responsiveness in ovarian cancer. This is important because it suggests that future combinations may not simply involve adding an IDO1 inhibitor to immunotherapy, but rather using more sophisticated approaches to reshape the broader metabolic architecture of the tumor microenvironment.

Overall, combination therapy remains one of the most promising routes for targeting tryptophan metabolism in cancer. However, the most effective combinations will likely depend on accurate patient stratification and a better understanding of which branches of the pathway are dominant in specific tumor contexts.

### 6.4 Targeting stromal and myeloid metabolic crosstalk

A particularly important future direction is the recognition that targeting tumor cells alone may not be sufficient. Because tryptophan metabolism operates across tumor, stromal, and immune compartments, therapy may need to disrupt the multicellular circuits that sustain immunosuppression (151, 152). Lu et al. showed that TDO2-positive CAFs impede CD8+ T-cell infiltration in cutaneous squamous cell carcinoma, directly implicating stromal metabolism in immune exclusion. Liu et al. similarly demonstrated that TDO2-positive matrix fibroblasts in metastatic breast cancer support both tumor survival and T-cell dysfunction. These findings suggest that stromal fibroblasts are not passive barriers but active participants in metabolic immune escape. Therapeutic strategies that fail to address these stromal contributors may leave major suppressive niches intact. The myeloid compartment is equally relevant. Xue et al. showed that tryptophan metabolism regulates macrophage polarization in breast cancer, while Zhao et al. linked TDO2 signaling to M2 macrophage polarization through the AKT/GSK3β/IL-8 axis in esophageal squamous cell carcinoma. Elomaa et al. further demonstrated spatial association between IDO/ARG1 expression and myeloid infiltration in colorectal cancer. Together, these studies indicate that tryptophan metabolism is deeply embedded in suppressive myeloid and stromal ecosystems. These observations point toward a broader therapeutic principle: future interventions may need to move from single-enzyme inhibition toward niche reprogramming. Rather than targeting only the tumor cell component of tryptophan metabolism, it may be necessary to dismantle the stromal and myeloid networks that maintain immune dysfunction and support metastatic adaptation.

### 6.5 Future directions

Several priorities should shape the next phase of research and therapeutic development in this field. First, more precise stratification is needed to determine which tumors are truly tryptophan-high and which patients are most likely to benefit from pathway-targeted intervention. Not all tumors with immune resistance will be equally dependent on this axis, and biomarker-guided selection will be essential. Second, future therapies should increasingly consider multi-branch intervention rather than focusing exclusively on kynurenine or a single catabolic enzyme. The growing recognition of pathway branching and compensatory flux suggests that broader rewiring of tryptophan metabolism may be required to reverse resistance effectively. Third, spatially resolved approaches such as spatial transcriptomics, multiplex imaging, and metabolomics should be integrated into future studies to define where suppressive tryptophan-metabolic niches are located and how they interact with immune and stromal populations. Such tools may identify lesion-specific vulnerabilities that are invisible in bulk analyses. Finally, tryptophan metabolism should be studied in closer connection with other adaptive processes, including ferroptosis resistance, metastatic niche formation, and nutrient competition within the tumor microenvironment. These interactions may reveal why the pathway is so effective in supporting tumor evolution and why it remains difficult to therapeutically disable.

Taken together, the future of this field lies in shifting from a narrow enzyme-centric view toward a systems-level understanding of tryptophan metabolism as a dynamic immunometabolic network. **Table 3** summarizes the current translational value of tryptophan metabolism, including biomarker development, immunotherapy response prediction, and emerging therapeutic strategies targeting this pathway.

**Table 3:** Translational significance and therapeutic opportunities of targeting tryptophan metabolism in cancer.

Translational aspect	Key finding	Clinical implication	Representative studies
Blood-based biomarker	Kyn/Trp ratio reflects altered tryptophan catabolism in NSCLC	Potential noninvasive biomarker for disease monitoring and stratification	Mandarano 2021 (132)
Immunotherapy response prediction	Increased tryptophan metabolism predicts pembrolizumab resistance in melanoma	May help identify patients less likely to respond to ICB alone	Oldan 2023 (138)
Myeloid-based prediction	Trp metabolism-related macrophage polarization correlates with breast cancer immunotherapy response	Suggests immunometabolic stratification beyond tumor-cell markers	Xue 2023 (88)
Exposure-linked immunotherapy resistance	Tobacco carcinogen induces IDO1 and immune suppression	Highlights exposure-related metabolic barriers to anti-PD-1 therapy	Liang 2022 (65)
Dual-enzyme targeting	Dual inhibition of IDO1/TDO2 enhances antitumor immunity in resistant NSCLC	Supports combined rather than single-node blockade	Wu 2023 (37)
Multi-branch metabolic rewiring	Dual inhibition of Kyn/indole and 5-HT branches restores ICB response in ovarian cancer	Suggests pathway-network targeting may outperform single-enzyme strategies	Li J 2024 (38)
Stromal targeting	TDO2+ CAFs and stromal fibroblasts promote immune exclusion and metastatic protection	Indicates stromal metabolic crosstalk as a therapeutic target	Lu 2025; Liu Y 2024 (39)
Spatial metabolic profiling	IDO/ARG1 programs co-localize with suppressive myeloid infiltration	Supports tissue-level and spatial biomarker development	Elomaa 2024 (70)

## 7 Conclusion

Tryptophan metabolism functions as an immunometabolic communication network that links tumor cells, stromal cells, and diverse immune populations to drive immune escape. Through effects on nutrient availability, kynurenine–AHR signaling, macrophage polarization, fibroblast-mediated niche remodeling, and CD8+ T-cell dysfunction, this pathway establishes a highly adaptable, immunosuppressive tumor microenvironment. Beyond classical immune suppression, tryptophan metabolism also contributes to ferroptosis resistance, stemness, metastatic colonization, and resistance to chemotherapy, targeted therapy, and immune checkpoint blockade. Translationally, circulating metabolites, tissue-level profiling, and spatially resolved analyses offer promising biomarker and patient stratification strategies. Therapeutically, single-node inhibition is unlikely to suffice; future strategies will require combination approaches, multi-branch metabolic intervention, and targeted disruption of stromal and myeloid metabolic crosstalk. Overall, understanding the integrated tryptophan metabolic network may guide more effective interventions to overcome tumor persistence and therapeutic resistance.
